# Ileal interposition reconstruction for ileo-rectal fistula following sex reassignment surgery: A case report

**DOI:** 10.1016/j.ijscr.2023.108523

**Published:** 2023-07-20

**Authors:** Nobuhiro Nitori, Tomoaki Deguchi, Ayu Kato, Fumihiko Kato, Masahiro Shinoda, Osamu Itano

**Affiliations:** a Center of Digestive Diseases, International University of Health and Welfare Mita Hospital, Mita 1-4-3, Minato-ku, Tokyo 108-8329, Japan; b Department of Surgery, Machida Hospital, Kiso-higasi 4-21-43, Machida-shi, Tokyo 194-0036, Japan; c Department of Hepato-Biliary-Pancreatic and Gastrointestinal Surgery, International University of Health and Welfare School of Medicine, 852 Hatakeda Narita, Chiba 286-0124, Japan

**Keywords:** Sigmoid vaginoplasty, Complication, Ileal interposition

## Abstract

**Introduction:**

Ileorectal fistulas following sigmoid colon vaginoplasty are rare. Reports on the management of the surgical complications of sex reassignment operations among transgender patients are few.

**Presentation of case:**

A 40-year-old patient with a male-to-female sex identity disorder underwent sigmoid vaginoplasty for sex reassignment 4 months prior to presentation. The patient was referred for persistent diarrhea and postoperative lower abdominal pain. Proctoscopy, gastrografin enema, and small bowel enterography revealed rectal anastomotic stenosis and an ileorectal fistula. The prior anastomotic site and ileal rectal fistula were resected, and ileal interposition reconstruction was performed to avoid damaging the blood supply to the artificial vagina. Routine follow-up after the closure of the diverting ileostomy showed no new pathologies.

**Discussion:**

This case highlighted the management of surgical complications after sex reassignment surgery.

**Conclusion:**

Ileal interposition was a useful reconstruction method after resecting the colonic anastomotic site to preserve the artificial vagina.

## Highlights

• An ileo-rectal fistula following vaginoplasty using sigmoid colon is rare.
• Care must be taken to avoid damaging the blood supply to the artificial vagina.
• An ileal interposition is useful for reconstruction.

## 1 Introduction

One approach in sex reassignment surgery (SRS) for transgender patients is vaginoplasty using the sigmoid colon. This procedure is reportedly safe with few complications, and the satisfaction with sexual life after surgery is high [1,2]. However, there have been few reports on managing its postoperative complications. This study reported a case of ileorectal fistula in a patient who previously underwent SRS. The work has been reported in line with the SCARE 2020 criteria [3].

## 2 Presentation of a case

A 40-year-old patient with a male-to-female gender identity disorder underwent SRS, involving bilateral orchiectomy, penectomy, clitoroplasty, vulvoplasty, and sigmoid vaginoplasty, 4 months ago in Thailand. Postoperatively, the patient had persistent diarrhea and occasional hypogastric pain. Plastic surgery of the thyroid cartilage was performed at the Otolaryngology Department in our hospital. While hospitalized, the patient was referred to the Center for Digestive Diseases for persistent diarrhea.

**Fig. 1:**
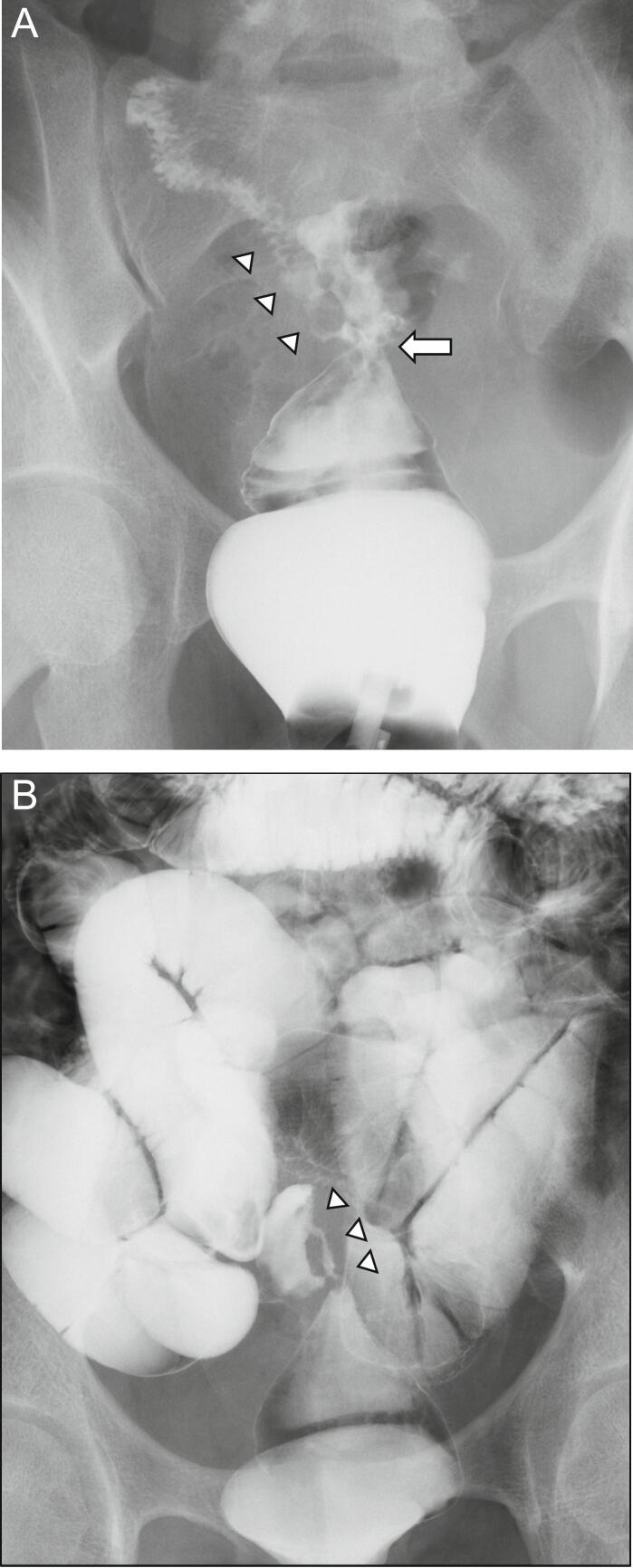
Gastrografin enema and enterography. Gastrografin enema (A) and small bowel enterography (B) showed rectal anastomotic stenosis (*arrow*) and an ileo-rectal fistula (*arrowhead*).

Proctoscopy revealed circumferential stenosis of the rectum. Gastrografin enema and small bowel enterography showed rectal anastomotic stenosis and an ileorectal fistula, indicating that the lumen of the stenosis was less than 1 cm and it was located in middle rectum within 10 cm from the anal verge. The length of the stenosis could not be assessed because almost all of the contrast material would flow through the fistula into the small intestine (Fig. 1A,B). During small bowel enterography, the contrast medium did not flow into the distal ileum and large intestine below the fistula site. However, it flowed into the rectum through the fistula. Based on the diagnosis of rectal anastomotic stenosis and small intestinal fistula, a laparotomy was performed on a semi-emergency basis.

**Fig. 2:**
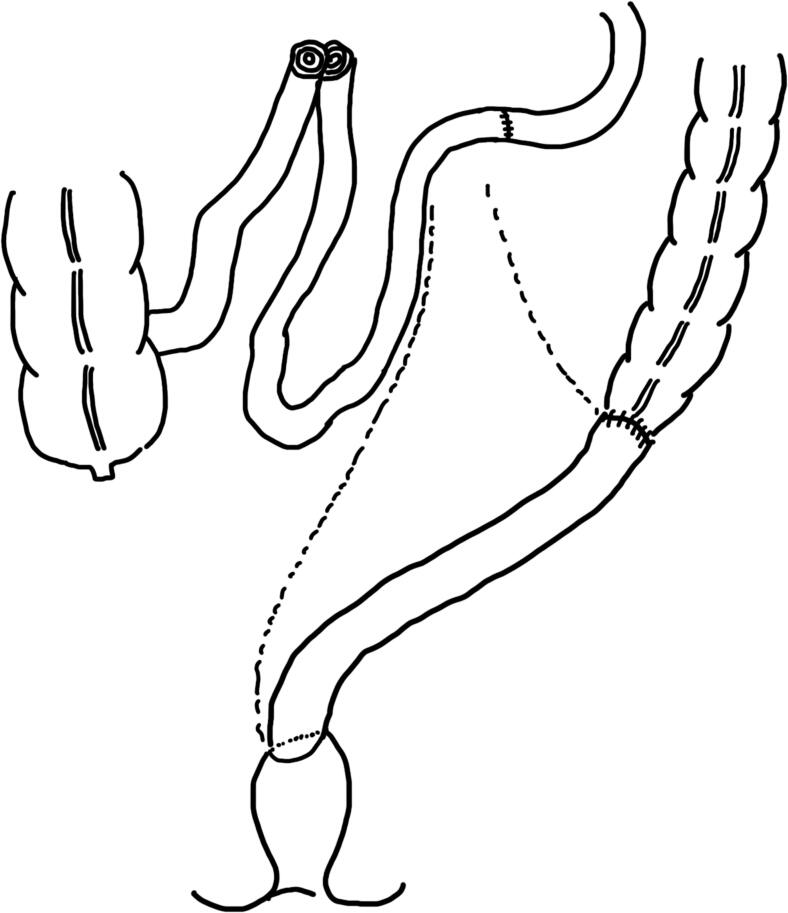
Schema of the ileal interposition. The ileo-rectal fistula was resected, and ileal interposition was performed.

The prior anastomotic site and ileal rectal fistula were resected; however, due to the adhesions following vaginal surgery, the rectum was not sufficiently detached. Since the artificial vagina was nourished by the inferior mesenteric artery, the mesentery of the previous anastomotic site was dissected along the intestinal wall to prevent damage to the mesenteric vasculature. Moreover, dehiscence of the mesenteric adhesion was not adequately achieved because the mesentery, which contained the blood supply of the artificial vagina, needed to be preserved. The descending colon did not reach the pelvis despite splenic flexure mobilization. Thus, an approximately 20 cm of ileal interposition reconstruction was performed, and a diverting ileostomy was constructed (Fig. 2). There were no specific pathologies in a resected specimen, including malignancies and inflammatory bowel diseases.

**Fig. 3:**
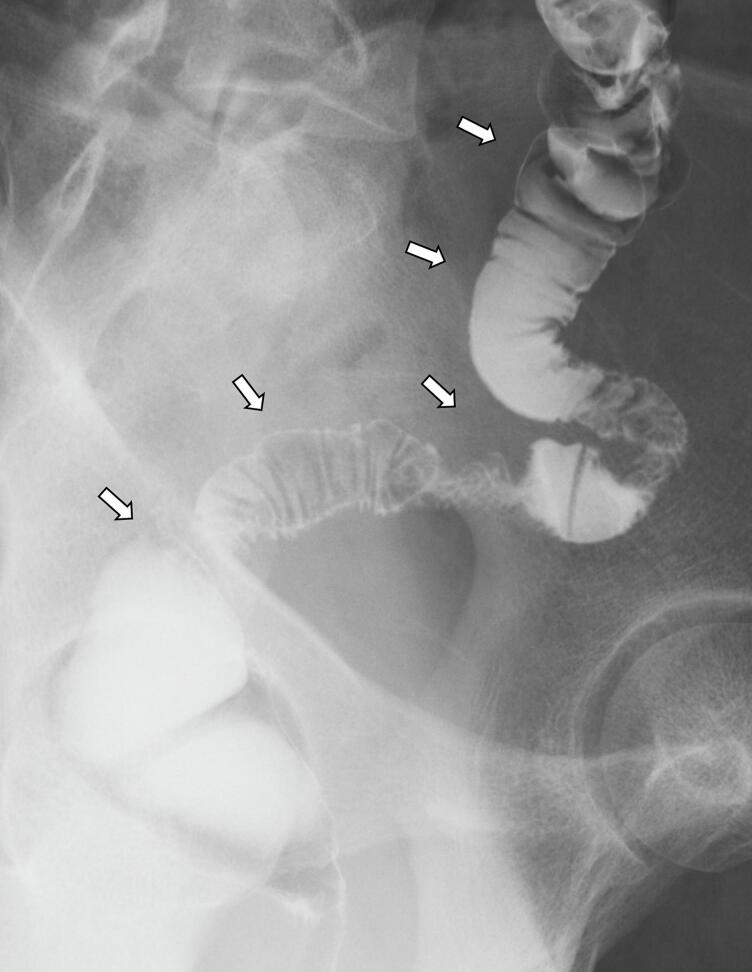
Postoperative gastrografin enema. The enema examination showed no suture failure and adequate passage through the interstitial ileum (*arrow*).

Three months later, the diverting ileostomy was closed because the enema examination showed no suture failure, and passage through the interstitial ileum was intact (Fig. 3). Routine follow-up after the ileostomy closure showed no new pathologies more than 10 years.

## 3 Discussion

To our knowledge, this is the first case report of ileo-rectal fistula following sex reassignment surgery (SRS). In this case, the previous anastomotic site was successfully resected without sacrificing the neovagina for an ileorectal fistula, which developed after SRS. The ileal interposition was an effective reconstruction modality because of the poor mobilization of the oral intestinal tract.

There are two main types of SRS for male-to-female transgender patients: the flap method, which uses a penile scrotal flap, and the large intestine method, which involves the sigmoid colon [4]. The advantages of using the sigmoid colon include good vaginal lubrication, adequate graft blood flow, and resistance to trauma [5]. Postoperative complications have been reported, including intralabial hematomas, perineal abscesses, urethral strictures, urinary incontinence, vaginal strictures, urethral vaginal fistulas, and rectal vaginal fistulas [6]. Complications were encountered in 6.4 % of patients who underwent vaginoplasty using the bowel [7]. Suture failure was an uncommon postoperative complication with an incidence of 2.4–7.7 %. However, it reportedly improved with fasting, decompression drainage, and a diverting ileostomy. There have been no reports of cases requiring re-excision of the anastomotic site. In the present case, the anastomotic leakage, anastomotic stenosis, and ileorectal fistula overlapped. Endoscopic stenting was not applicable for stenosis in benign diseases in Japan at that time. Moreover, during small bowel enterography, the contrast medium did not flow into the distal ileum and large intestine below the fistula site; thus, excision and re-anastomosis were warranted. Anastomotic resection after sigmoid vaginoplasty is difficult because the blood flow to the artificial vagina should be maintained. Ileal interposition was selected because the small intestine proximal to the ileorectal fistula had adequate mobility, which contributed to the success of the procedure.

An alternative method is an ileocecal interposition, as described by von Flue and Harder [8]. The pedunculated ileocecal segment is rotated 180° counterclockwise and placed between the descending colon and the rectum. When applying this method to the present case, four anastomotic sites are required; hence, the procedure is more complicated. However, in cases involving a long defect, transferring the right colon allows it to be placed over a longer distance. Although Cattel Braasch maneuver is useful in mobilization of all the colon, the blood supply of the artificial vagina crucially needed to be preserved in the present case; thus we did not try that maneuver.

In the present case, the prior anastomotic stenosis was caused by ischemia of the colon proximal to the anastomosis site, based on the circumferential stenosis over the distance above the anastomotic site. Studies on the benefits of indocyanine green (ICG) fluorescence in colorectal surgery suggest that the procedure is preferred to avoid anastomotic leakage despite little scientific evidence [9,10]. Moreover, in SRS, the sigmoidal artificial vagina must be pulled maximally towards the pelvis, while monitoring for intestinal ischemia. Assessing bowel circulation with ICG was unavailable despite being more common now than it was 15 years ago, when SRS and the second operation were performed. Flor-Lorente et al. have recently reported that laparoscopic sigmoid vaginoplasty under ICG fluorescence guidance enabled the visualization of the sigmoid and neovaginal vasculature, thereby facilitating selective ligation of vessels [11]. They concluded that the complications should be minimized when using new technology, such as ICG.

Finally, the overall number of patients with gender dysphoria has reportedly increased [12]. Consequently, the number of SRS-es is expected to increase in the future. Therefore, it is crucial to know how to manage its complications.

## 4 Conclusion

Ileal interposition was a useful reconstruction technique after resecting the colonic anastomotic site to preserve the artificial vagina in a patient who underwent SRS.

## Declaration of competing interest

None of the authors had conflicts of interests associated with this case report.

## Data Availability

All data generated or analyzed in the present study were included in this published article. Further inquiries may be directed to the corresponding author.
